# Aquabis[1-hydroxy-2-(imidazol-3-ium-1-yl)-1,1′-ethylidenediphophonato-κ^2^ *O*,*O*′]zinc(II) dihydrate

**DOI:** 10.1107/S160053680904286X

**Published:** 2009-10-23

**Authors:** Eleonora Freire, Daniel R. Vega

**Affiliations:** a Gerencia de Investigación y Aplicaciones, Centro Atómico Constituyentes, Comisión Nacional de Energía Atómica and, Escuela de Ciencia y Tecnología, Universidad Nacional General San Martín, Buenos Aires, Argentina

## Abstract

In the title complex, [Zn(C_5_H_9_NO_7_P_2_)_2_(H_2_O)]·2H_2_O, the zinc atom is coordinated by two zoledronate anions  [zoledronate = (2-(1-imidazole)-1-hydr­oxy-1,1′-ethyl­idenediphophonate)] and one water mol­ecule. The coordination number is 5. There is one half-mol­ecule in the asymmetric unit, the zinc atom being located on a twofold rotation axis passing through the metal centre and the coordinating water O atom. The anion exists as a zwitterion with an overall charge of −1; the protonated nitro­gen in the ring has a positive charge and the two phospho­nates groups each have a single negative charge. Inter­molecular O—H⋯O hydrogen bonds link the mol­ecules. An N—H⋯O inter­action is also present.

## Related literature

For general background to bis­phospho­nates, see: Fleisch *et al.* (1968 ▶); Green *et al.* (1994 ▶); Fleisch (2000 ▶); Ross *et al.* (2004 ▶); Smith (2005 ▶); Ralston *et al.* (1989 ▶); Reid *et al.* (2005 ▶); Rauch & Glorieux (2005 ▶); Chesnut *et al.* (2004 ▶). For structures of transition metal (Ni, Co and Cu) complexes with the zoledronate anion, see: Cao *et al.* (2007 ▶, 2008 ▶). For metal complexes of other bis­phospho­nates (Etidronate and Pamidronate), see: Fernández *et al.* (2002 ▶); Li *et al.* (2008 ▶); Chen *et al.* (2008 ▶); Uchtman (1972 ▶). For a hexa­coordinated zinc(II)–zoledronate complex, see: Freire & Vega (2009 ▶).
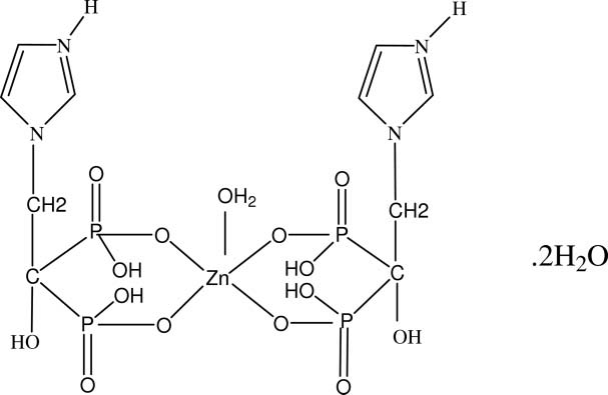


## Experimental

### 

#### Crystal data

[Zn(C_5_H_9_N_2_O_7_P_2_)_2_(H_2_O)]·2H_2_O
*M* *_r_* = 661.58
Monoclinic, 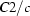

*a* = 12.089 (2) Å
*b* = 9.858 (2) Å
*c* = 18.831 (4) Å
β = 95.09 (3)°
*V* = 2235.3 (8) Å^3^
*Z* = 4
Mo *K*α radiation
μ = 1.48 mm^−1^
*T* = 293 K
0.20 × 0.18 × 0.09 mm

#### Data collection

Rigaku AFC6 diffractometer
Absorption correction: ψ scan (North *et al.*, 1968 ▶) *T*_min_ = 0.75, *T*_max_ = 0.87
2847 measured reflections
2208 independent reflections
1528 reflections with *I* > 2σ(*I*)
*R*_int_ = 0.054
3 standard reflections every 150 reflections intensity decay: <3%

#### Refinement

*R*[*F* ^2^ > 2σ(*F* ^2^)] = 0.032
*wR*(*F* ^2^) = 0.096
*S* = 1.00
2208 reflections
167 parameters
H-atom parameters constrained
Δρ_max_ = 0.35 e Å^−3^
Δρ_min_ = −0.87 e Å^−3^

### 

Data collection: *MSC/AFC Diffractometer Control Software* (Molecular Structure Corporation, 1988 ▶); cell refinement: *MSC/AFC Diffractometer Control Software*; data reduction: *MSC/AFC Diffractometer Control Software*; program(s) used to solve structure: *SHELXS97* (Sheldrick, 2008 ▶); program(s) used to refine structure: *SHELXL97* (Sheldrick, 2008 ▶); molecular graphics: *SHELXTL* (Sheldrick, 2008 ▶); software used to prepare material for publication: *SHELXTL* and *PLATON* (Spek, 2009 ▶).

## Supplementary Material

Crystal structure: contains datablocks global, I. DOI: 10.1107/S160053680904286X/bq2166sup1.cif

Structure factors: contains datablocks I. DOI: 10.1107/S160053680904286X/bq2166Isup2.hkl

Additional supplementary materials:  crystallographic information; 3D view; checkCIF report

## Figures and Tables

**Table:** 

Zn1—O1*W*	1.999 (4)
Zn1—O11	2.006 (2)
Zn1—O21	2.041 (2)

**Table:** 

O1*W*—Zn1—O11	112.97 (8)
O11—Zn1—O11^i^	134.06 (15)
O11—Zn1—O21^i^	89.03 (9)
O1*W*—Zn1—O21	88.48 (7)
O11—Zn1—O21	92.16 (9)
O21^i^—Zn1—O21	176.96 (14)

Symmetry code: (i) 
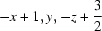
.

**Table 2:** Hydrogen-bond geometry (Å, °)

*D*—H⋯*A*	*D*—H	H⋯*A*	*D*⋯*A*	*D*—H⋯*A*
O12—H12⋯O2*W*	0.82	1.75	2.566 (3)	171
O22—H22⋯O13^ii^	0.82	1.84	2.645 (3)	167
O2*W*—H2*WA*⋯O21^iii^	0.82	2.38	2.990 (3)	132
O2*W*—H2*WB*⋯O13^iv^	0.82	1.94	2.758 (4)	173
N2—H2⋯O12^v^	0.86	2.11	2.917 (4)	157
O1*W*—H1*W*⋯O23^ii^	0.82	1.81	2.632 (3)	177
O1—H1⋯O23^ii^	0.82	1.80	2.581 (3)	160

Symmetry codes: (ii) 
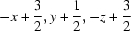
; (iii) 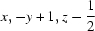; (iv) 
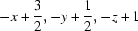
; (v) 
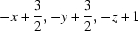
.

**Fig. 1.**
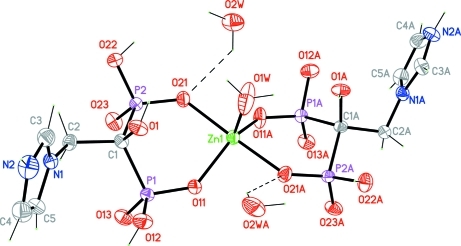
: Molecular view of (I), showing the labeling scheme used. Hydrogen bonding is shown in dashed lines.

**Fig. 2.**
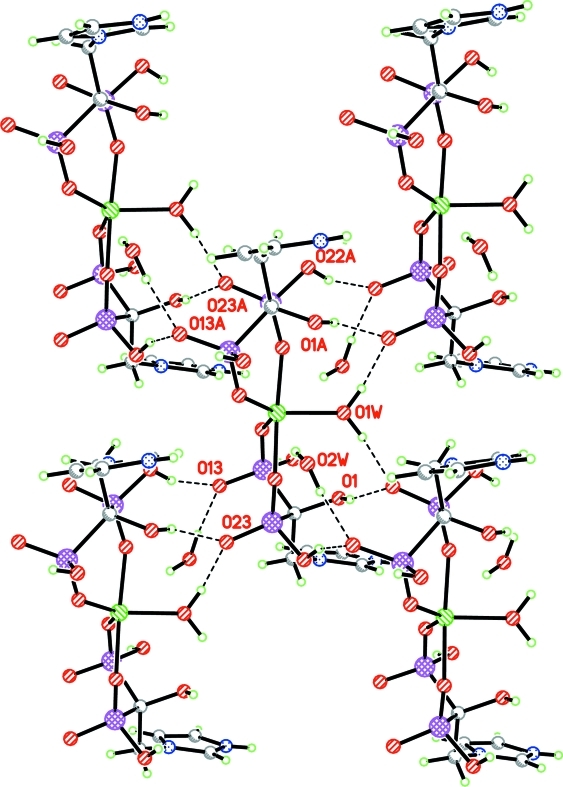
: Full packing diagram of (I).

## supplementary crystallographic information

### Comment

The following work is part of a project directed to the preparation and characterization of coordination complexes obtained by the interaction among metals and organic molecules of relevant pharmacological interest like bisphosphonates. An informative introduction on bisphosphonates has been made in the previous paper (Freire & Vega, 2009). Although few metal derivatives of Zoledronic acid have been reported in CSD (Allen, 2002), an isostructural compound of copper has been synthesized (Cao *et al.*, 2008).

So, we present herein the crystal structure of a Zinc-Zoledronate complex: monozinc dizoledronate trihydrate, (I), Zn.(H_2_O).2(P_2_O_7_N_2_C_5_H_9_).2H_2_O. In (I), as in the similar hexacoordinated compound (Freire & Vega, 2009), the zoledronate anion exists as a zwitterion with an overall charge of -1; the protonated nitrogen in the ring has a positive charge and the two phosphonates groups each have a single negative charge.

The coordination number of Zn is 5 (Fig. 1) and the resulting coordination polyhedron is a trigonal bipiramid defined by O21, O21A, O11, O11A and O1W. Atoms O11A and O21A are generated by the symmetry operation (1 - *x*, *y*, 3/2 - *z*). The equatorial plane is defined by O11, O11A and O1W, the apexes are defined by and O21 and O21A. The apical Zn—O distance is 2.041 (2) Å while in the equatorial plane the mean value for the Zn—O distance is 2.004 (4) Å. The coordination angles in the equatorial plane are a little turned aside from the expected 120 ° theoretically due to the "bite" of the ligand: O11—Zn—O11A 134.08 (15)°, O11—Zn—O1W and O11A—Zn—O1W are 112.96 (8) °. The angle between the line defined by O21 and O21A with the normal to the equatorial plane (O11, O11A, O1W and Zn1) is 2.3 °.

Considering the bisphosphonates groups, there are two distinct types of P—O bonds, as shown by the mean value in the following bond distances and angles: P—OH 1,576 (8), P - O 1.505 (5) Å, O—P—OH 109.4° (11), O—P—O 116.4° (14). The staggered conformation of PO3 groups in compound (II) is more prominent than in the hexacoordinated complex (Freire & Vega, 2009), the non bonded torsion angle O12—P1···P2 O22 is -16.1°. In (I), the imidazol ring is planar, maximum deviation from the *L*. S. mean plane is 0.0026 Å for C3, and it is not coplanar with C2, between the plane of the ring and the bond N1—C2 is 3.4 ° and C2 is 0.0837 Å far from the ring. The torsion angle C1—C2—N1—C3 is of -78.62 ° and it is possible to describe it like - Syn-Clinal (-*sc*).

Five hydrogen bonds, involving, H22, H1W, H1, H2WA and H2WB, provide intermolecular cohesion, defining a two-dimensional arrangement, while the three-dimensional net completes with two more hydrogen bonds, involving H2 from the aromatic ring and H12 from the bisphosphonate group (Fig. 2 and Table 2).

### Experimental

Zoledronic Acid was obtained from Gador S. A. laboratory. Compound (II) was obtained by direct mix of a water solution of Zoledronic Acid and a water solution of ZnCl_2_. Colorless prismatic crystals were grown after a few days.

### Refinement

The H atoms attached to O were found in a difference Fourier map, further idealized (O—H: 0.82 Å - 0.90 Å) and finally allowed to ride. Those attached to C and N were placed at calculated positions (C—H: 0.93 Å; C—H_2_: 0.97 Å; N—H_2_: 0.90 Å) and allowed to ride. Displacement factors were taken as U(H)_isot_ = *x*.U(host), *x*: 1.2 (C—H); 1.5 (C—H_2_, N—H_2_, O—H).

### Crystal data

**Table:**

[Zn(C_5_H_9_N_2_O_7_P_2_)_2_(H_2_O)]·2H_2_O	*F*(000) = 1352
*M**_r_* = 661.58	*D*_x_ = 1.966 Mg m^−^^3^
Monoclinic, *C*2/*c*	Mo *K*α radiation, λ = 0.71073 Å
Hall symbol: -C 2yc	Cell parameters from 42 reflections
*a* = 12.089 (2) Å	θ = 8–25°
*b* = 9.858 (2) Å	µ = 1.48 mm^−^^1^
*c* = 18.831 (4) Å	*T* = 293 K
β = 95.09 (3)°	Prism, colorless
*V* = 2235.3 (8) Å^3^	0.20 × 0.18 × 0.09 mm
*Z* = 4	

### Data collection

**Table:**

Rigaku AFC6 diffractometer	1528 reflections with *I* > 2σ(*I*)
Radiation source: fine-focus sealed tube	*R*_int_ = 0.054
graphite	θ_max_ = 26.0°, θ_min_ = 2.2°
ω/2θ scans	*h* = −1→14
Absorption correction: ψ scan (North *et al.*, 1968)	*k* = −1→12
*T*_min_ = 0.75, *T*_max_ = 0.87	*l* = −23→23
2847 measured reflections	3 standard reflections every 150 reflections
2208 independent reflections	intensity decay: <3%

### Refinement

**Table:**

Refinement on *F*^2^	Primary atom site location: structure-invariant direct methods
Least-squares matrix: full	Secondary atom site location: difference Fourier map
*R*[*F*^2^ > 2σ(*F*^2^)] = 0.032	Hydrogen site location: inferred from neighbouring sites
*wR*(*F*^2^) = 0.096	H-atom parameters constrained
*S* = 1.00	*w* = 1/[σ^2^(*F*_o_^2^) + (0.0521*P*)^2^ + 1.3378*P*] where *P* = (*F*_o_^2^ + 2*F*_c_^2^)/3
2208 reflections	(Δ/σ)_max_ = 0.001
167 parameters	Δρ_max_ = 0.35 e Å^−^^3^
0 restraints	Δρ_min_ = −0.87 e Å^−^^3^

### Special details

**Table:**

Geometry. All e.s.d.'s (except the e.s.d. in the dihedral angle between two l.s. planes) are estimated using the full covariance matrix. The cell e.s.d.'s are taken into account individually in the estimation of e.s.d.'s in distances, angles and torsion angles; correlations between e.s.d.'s in cell parameters are only used when they are defined by crystal symmetry. An approximate (isotropic) treatment of cell e.s.d.'s is used for estimating e.s.d.'s involving l.s. planes.
Refinement. Refinement of *F*^2^ against ALL reflections. The weighted *R*-factor *wR* and goodness of fit *S* are based on *F*^2^, conventional *R*-factors *R* are based on *F*, with *F* set to zero for negative *F*^2^. The threshold expression of *F*^2^ > σ(*F*^2^) is used only for calculating *R*-factors(gt) *etc*. and is not relevant to the choice of reflections for refinement. *R*-factors based on *F*^2^ are statistically about twice as large as those based on *F*, and *R*- factors based on ALL data will be even larger.

### Fractional atomic coordinates and isotropic or equivalent isotropic displacement parameters (Å^2^)

**Table:**

	*x*	*y*	*z*	*U*_iso_*/*U*_eq_	
Zn1	0.5000	0.50532 (5)	0.7500	0.02015 (15)	
P1	0.64482 (6)	0.41327 (8)	0.61862 (4)	0.01916 (19)	
P2	0.76934 (6)	0.48891 (8)	0.75923 (4)	0.01749 (19)	
O1	0.71512 (19)	0.6590 (2)	0.64590 (11)	0.0237 (5)	
H1	0.7154	0.7093	0.6806	0.043 (12)*	
O11	0.54208 (18)	0.4259 (3)	0.65807 (12)	0.0299 (5)	
O12	0.6205 (2)	0.4826 (2)	0.54319 (13)	0.0288 (5)	
H12	0.6271	0.4369	0.5075	0.050 (14)*	
O13	0.68721 (19)	0.2707 (2)	0.61059 (12)	0.0277 (5)	
O21	0.66152 (17)	0.5108 (2)	0.79201 (11)	0.0253 (5)	
O22	0.85809 (18)	0.5945 (2)	0.79009 (12)	0.0269 (5)	
H22	0.8339	0.6491	0.8177	0.051 (14)*	
O23	0.81904 (19)	0.3498 (2)	0.76479 (12)	0.0280 (5)	
O1W	0.5000	0.7080 (4)	0.7500	0.0727 (17)	
H1W	0.5565	0.7530	0.7470	0.109*	
O2W	0.6191 (2)	0.3463 (3)	0.42704 (13)	0.0394 (6)	
H2WA	0.5899	0.3910	0.3937	0.059*	
H2WB	0.6790	0.3124	0.4196	0.059*	
N1	0.8788 (2)	0.5706 (3)	0.56618 (13)	0.0218 (5)	
N2	0.8895 (2)	0.7275 (3)	0.48801 (15)	0.0328 (7)	
H2	0.8972	0.8060	0.4691	0.039*	
C1	0.7536 (2)	0.5248 (3)	0.66243 (16)	0.0183 (6)	
C2	0.8707 (2)	0.5064 (3)	0.63530 (16)	0.0216 (6)	
H2A	0.9262	0.5453	0.6698	0.026*	
H2B	0.8865	0.4103	0.6315	0.026*	
C3	0.8930 (3)	0.7035 (3)	0.55705 (18)	0.0278 (7)	
H3	0.9034	0.7680	0.5931	0.033*	
C4	0.8718 (3)	0.6092 (4)	0.45095 (19)	0.0351 (8)	
H4	0.8657	0.5991	0.4017	0.042*	
C5	0.8649 (3)	0.5096 (4)	0.49990 (17)	0.0282 (7)	
H5	0.8530	0.4178	0.4907	0.034*	

### Atomic displacement parameters (Å^2^)

**Table:**

	*U*^11^	*U*^22^	*U*^33^	*U*^12^	*U*^13^	*U*^23^
Zn1	0.0189 (2)	0.0212 (3)	0.0206 (3)	0.000	0.00281 (18)	0.000
P1	0.0201 (4)	0.0188 (4)	0.0187 (4)	−0.0007 (3)	0.0023 (3)	−0.0026 (3)
P2	0.0181 (4)	0.0171 (4)	0.0172 (4)	0.0010 (3)	0.0010 (3)	0.0012 (3)
O1	0.0348 (12)	0.0148 (10)	0.0211 (11)	0.0063 (9)	−0.0003 (9)	0.0005 (9)
O11	0.0207 (11)	0.0397 (14)	0.0304 (13)	−0.0072 (10)	0.0080 (9)	−0.0142 (11)
O12	0.0363 (13)	0.0275 (12)	0.0217 (11)	0.0036 (10)	−0.0033 (10)	−0.0023 (10)
O13	0.0378 (13)	0.0175 (11)	0.0282 (11)	0.0015 (10)	0.0044 (10)	−0.0035 (10)
O21	0.0190 (10)	0.0362 (13)	0.0208 (11)	0.0021 (9)	0.0029 (8)	−0.0011 (10)
O22	0.0231 (11)	0.0291 (13)	0.0284 (12)	−0.0052 (9)	0.0019 (9)	−0.0081 (11)
O23	0.0360 (13)	0.0193 (11)	0.0296 (12)	0.0064 (9)	0.0072 (10)	0.0062 (9)
O1W	0.025 (2)	0.0195 (19)	0.177 (6)	0.000	0.027 (3)	0.000
O2W	0.0535 (17)	0.0414 (15)	0.0233 (12)	0.0094 (13)	0.0041 (11)	−0.0019 (11)
N1	0.0212 (12)	0.0244 (13)	0.0203 (13)	−0.0008 (10)	0.0051 (10)	−0.0006 (12)
N2	0.0363 (16)	0.0302 (15)	0.0324 (15)	−0.0009 (12)	0.0062 (13)	0.0132 (14)
C1	0.0207 (14)	0.0144 (13)	0.0197 (14)	0.0035 (11)	0.0020 (11)	0.0013 (11)
C2	0.0193 (14)	0.0239 (16)	0.0217 (15)	−0.0002 (12)	0.0025 (12)	0.0034 (13)
C3	0.0288 (17)	0.0252 (16)	0.0304 (18)	−0.0040 (13)	0.0073 (13)	0.0004 (14)
C4	0.0333 (19)	0.049 (2)	0.0230 (17)	0.0047 (16)	0.0012 (14)	0.0041 (17)
C5	0.0291 (17)	0.0322 (18)	0.0237 (16)	−0.0001 (14)	0.0044 (13)	−0.0070 (15)

### Geometric parameters (Å, °)

**Table:**

Zn1—O1W	1.999 (4)	O1W—H1W	0.8200
Zn1—O11	2.006 (2)	O2W—H2WA	0.8200
Zn1—O11^i^	2.006 (2)	O2W—H2WB	0.8200
Zn1—O21^i^	2.041 (2)	N1—C3	1.335 (4)
Zn1—O21	2.041 (2)	N1—C5	1.382 (4)
P1—O13	1.508 (2)	N1—C2	1.458 (4)
P1—O11	1.508 (2)	N2—C3	1.318 (4)
P1—O12	1.580 (2)	N2—C4	1.366 (5)
P1—C1	1.851 (3)	N2—H2	0.8600
P2—O23	1.497 (2)	C1—C2	1.558 (4)
P2—O21	1.506 (2)	C2—H2A	0.9700
P2—O22	1.569 (2)	C2—H2B	0.9700
P2—C1	1.850 (3)	C3—H3	0.9300
O1—C1	1.427 (3)	C4—C5	1.355 (5)
O1—H1	0.8200	C4—H4	0.9300
O12—H12	0.8200	C5—H5	0.9300
O22—H22	0.8200		
			
O1W—Zn1—O11	112.97 (8)	H2WA—O2W—H2WB	114.6
O1W—Zn1—O11^i^	112.97 (7)	C3—N1—C5	108.5 (3)
O11—Zn1—O11^i^	134.06 (15)	C3—N1—C2	124.1 (3)
O1W—Zn1—O21^i^	88.48 (7)	C5—N1—C2	127.2 (3)
O11—Zn1—O21^i^	89.03 (9)	C3—N2—C4	109.9 (3)
O11^i^—Zn1—O21^i^	92.16 (9)	C3—N2—H2	125.0
O1W—Zn1—O21	88.48 (7)	C4—N2—H2	125.0
O11—Zn1—O21	92.16 (9)	O1—C1—C2	108.9 (2)
O11^i^—Zn1—O21	89.03 (9)	O1—C1—P2	113.3 (2)
O21^i^—Zn1—O21	176.96 (14)	C2—C1—P2	106.45 (19)
O13—P1—O11	115.37 (14)	O1—C1—P1	104.41 (18)
O13—P1—O12	110.54 (13)	C2—C1—P1	114.5 (2)
O11—P1—O12	108.12 (14)	P2—C1—P1	109.42 (15)
O13—P1—C1	111.36 (13)	N1—C2—C1	112.1 (2)
O11—P1—C1	108.35 (13)	N1—C2—H2A	109.2
O12—P1—C1	102.23 (13)	C1—C2—H2A	109.2
O23—P2—O21	117.33 (14)	N1—C2—H2B	109.2
O23—P2—O22	108.95 (14)	C1—C2—H2B	109.2
O21—P2—O22	109.95 (13)	H2A—C2—H2B	107.9
O23—P2—C1	104.47 (13)	N2—C3—N1	108.1 (3)
O21—P2—C1	111.03 (13)	N2—C3—H3	126.0
O22—P2—C1	104.20 (13)	N1—C3—H3	126.0
C1—O1—H1	114.1	C5—C4—N2	106.7 (3)
P1—O11—Zn1	137.76 (14)	C5—C4—H4	126.6
P1—O12—H12	118.4	N2—C4—H4	126.6
P2—O21—Zn1	132.07 (13)	C4—C5—N1	106.8 (3)
P2—O22—H22	113.5	C4—C5—H5	126.6
Zn1—O1W—H1W	122.7	N1—C5—H5	126.6
			
O13—P1—O11—Zn1	114.0 (2)	O13—P1—C1—O1	162.06 (18)
O12—P1—O11—Zn1	−121.6 (2)	O11—P1—C1—O1	−70.0 (2)
C1—P1—O11—Zn1	−11.6 (3)	O12—P1—C1—O1	44.0 (2)
O1W—Zn1—O11—P1	70.5 (2)	O13—P1—C1—C2	43.0 (2)
O11^i^—Zn1—O11—P1	−109.5 (2)	O11—P1—C1—C2	170.9 (2)
O21^i^—Zn1—O11—P1	158.5 (2)	O12—P1—C1—C2	−75.0 (2)
O21—Zn1—O11—P1	−18.7 (2)	O13—P1—C1—P2	−76.38 (17)
O23—P2—O21—Zn1	−95.2 (2)	O11—P1—C1—P2	51.53 (18)
O22—P2—O21—Zn1	139.62 (18)	O12—P1—C1—P2	165.57 (14)
C1—P2—O21—Zn1	24.8 (2)	C3—N1—C2—C1	78.5 (4)
O1W—Zn1—O21—P2	−102.54 (19)	C5—N1—C2—C1	−96.1 (3)
O11—Zn1—O21—P2	10.4 (2)	O1—C1—C2—N1	−39.8 (3)
O11^i^—Zn1—O21—P2	144.5 (2)	P2—C1—C2—N1	−162.3 (2)
O23—P2—C1—O1	−175.2 (2)	P1—C1—C2—N1	76.6 (3)
O21—P2—C1—O1	57.4 (2)	C4—N2—C3—N1	0.6 (4)
O22—P2—C1—O1	−60.9 (2)	C5—N1—C3—N2	−0.6 (4)
O23—P2—C1—C2	−55.5 (2)	C2—N1—C3—N2	−176.1 (3)
O21—P2—C1—C2	177.09 (19)	C3—N2—C4—C5	−0.3 (4)
O22—P2—C1—C2	58.8 (2)	N2—C4—C5—N1	0.0 (4)
O23—P2—C1—P1	68.72 (17)	C3—N1—C5—C4	0.4 (4)
O21—P2—C1—P1	−58.67 (18)	C2—N1—C5—C4	175.7 (3)
O22—P2—C1—P1	−176.99 (13)		

Symmetry codes: (i) −*x*+1, *y*, −*z*+3/2.

### Hydrogen-bond geometry (Å, °)

**Table:**

*D*—H···*A*	*D*—H	H···*A*	*D*···*A*	*D*—H···*A*
O12—H12···O2W	0.82	1.75	2.566 (3)	171
O22—H22···O13^ii^	0.82	1.84	2.645 (3)	167
O2W—H2WA···O21^iii^	0.82	2.38	2.990 (3)	132
O2W—H2WB···O13^iv^	0.82	1.94	2.758 (4)	173
N2—H2···O12^v^	0.86	2.11	2.917 (4)	157
O1W—H1W···O23^ii^	0.82	1.81	2.632 (3)	177
O1—H1···O23^ii^	0.82	1.80	2.581 (3)	160

Symmetry codes: (ii) −*x*+3/2, *y*+1/2, −*z*+3/2; (iii) *x*, −*y*+1, *z*−1/2; (iv) −*x*+3/2, −*y*+1/2, −*z*+1; (v) −*x*+3/2, −*y*+3/2, −*z*+1.
